# Open Genetic Code: on open source in the life sciences

**DOI:** 10.1186/2195-7819-10-2

**Published:** 2014-01-09

**Authors:** Eric Deibel

**Affiliations:** Department of Biotechnology, Delft University of Technology, Kragujevac, The Netherlands; Institute Francilien Recherche, Innovation Société (IFRIS), Paris, France

**Keywords:** Open source, Life sciences, Informatics, Synthetic biology, Patents, Genetic engineering

## Abstract

The introduction of open source in the life sciences is increasingly being suggested as an alternative to patenting. This is an alternative, however, that takes its shape at the intersection of the life sciences and informatics. Numerous examples can be identified wherein open source in the life sciences refers to access, sharing and collaboration as informatic practices. This includes open source as an experimental model and as a more sophisticated approach of genetic engineering. The first section discusses the greater flexibly in regard of patenting and the relationship to the introduction of open source in the life sciences. The main argument is that the ownership of knowledge in the life sciences should be reconsidered in the context of the centrality of DNA in informatic formats. This is illustrated by discussing a range of examples of open source models. The second part focuses on open source in synthetic biology as exemplary for the re-materialization of information into food, energy, medicine and so forth. The paper ends by raising the question whether another kind of alternative might be possible: one that looks at open source as a model for an alternative to the commodification of life that is understood as an attempt to comprehensively remove the restrictions from the usage of DNA in any of its formats.

## Introduction

The title of this article – open genetic code – refers to the introduction of open source in the life sciences. The analogy is straightforward: open source is about the free availability of information, which includes information on genes, proteins, cells and so forth. What such a definition leaves unexamined, however, is its interpretation of 'free' and 'open' at the intersection of the life sciences and informatics. What does such an alternative to patenting mean when moving from examples that are directly equivalent to informatics and the response by the open source movement to the commodification of source code to the life sciences where markets and technologies are transforming the usage of DNA?

**Figure 1:**
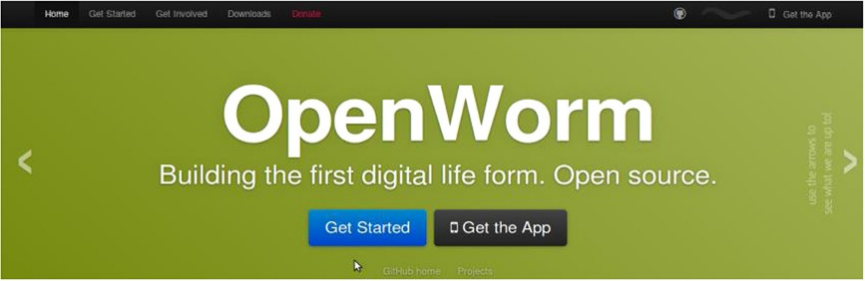
**Image taken from the website of the OpenWorm project on the 13th of May 2013, see** http://www.openworm.org/.

**Figure 2:**
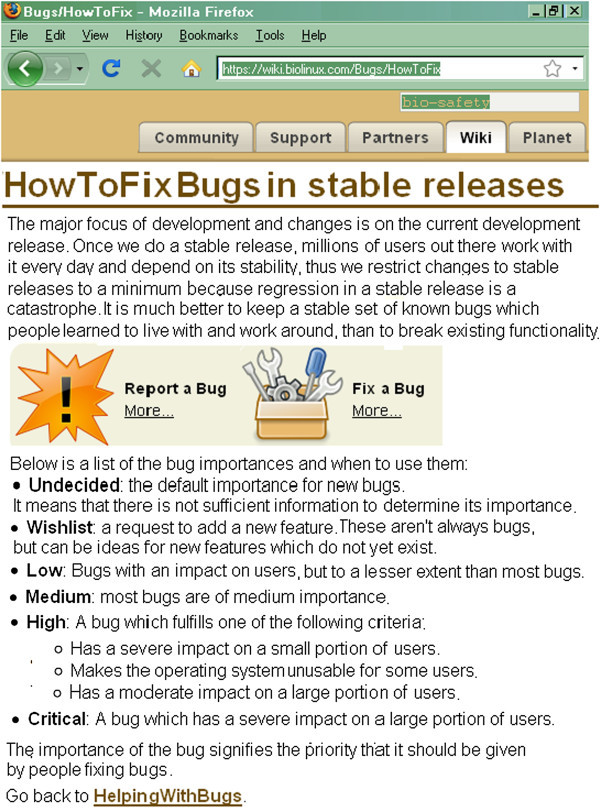
**Adapted by the author on the basis of** https://wiki.ubuntu.com/Bugs/HowToFix **, last checked may 2013.**

The suggestion that open source in the life sciences is an alternative to patenting in the life sciences typically refers to an attempt to solve the *tragedy of the anticommons.* What is tragic is than a situation wherein there are too many parties hold exclusive rights over access that are obstructing the usage of technologies, resources and the production of knowledge (Heller 1998 Heller and Eisenberg 1998). To solve the tragedy requires that the scope of patenting is limited by supporting a greater extend of “openness” in the life sciences, which takes the shape of trading and cross-licensing of patents as well as the arrangement of access by way of collaborative research projects, joint ventures, private consortia and public–private partnerships (see Allarakhia 2007). Most commentators have interpreted the introduction of open source in the life sciences along these lines: as a response to the proliferation of patents analogous to the example set by the open source movement in informatics (see Burk 2002; Reichman and Uhlir 2003; Boettiger and Burk 2004; Opderbeck 2004; Hope 2008,2009; Overwalle 2009; Allarakhia and Wensley 2005,2007; Rai & Boyle 2007).

However, there are various types of resources and knowledge in the life sciences that have become market-based over the last decades. Crucially, this includes the values of openness and collaboration in the life sciences, which are not necessarily the attributes of academic projects wherein commercial imperatives have no place (Kleinman and Vallas 2006). Openness in the life sciences as an increasingly regulated and commercialized activity represents a considerable strategic and economic value when situating the decision not to pursue patent strategies within the context of a 'contestation over the very definition of what constitutes market logic' and in terms of its 'implications for the overall terrain of cooperation, conflict and value generation' in the life sciences (Sunder Rajan 2006 Sunder Rajan 2003). This does not imply that that the introduction of open source in the life sciences does not have any potential as a 'defense of the public domain' or as a new kind of commons that is noncommercial (see Lessing 2001 Benkler 2002,2006 see Boyle [Bibr CR12][Bibr CR13][Bibr CR14]). However, there should be little surprise about how many of the alternatives to patenting resemble an organizational setting wherein innovation involves start-up companies, venture capital, stock markets and other kinds of financial speculation (see Cooper 2008).

The first section discusses the greater flexibly in regard of patenting and the relationship to the introduction of open source in the life sciences. The main argument is that the ownership of knowledge should be reconsidered in relation to the centrality of DNA in informatic formats. A range of examples of examples of open source models illustrate the convergence with informatics and show an alternative that takes shape as informatic practices, artifacts and related sets of knowledge in the life sciences. The objective of this part of the analysis is to turn the discussion away from a juridical understanding of openness that remains closely tied to patenting and towards an analytical position in respect of the redefinition of openness in the life sciences. Accordingly the main argument of section one is that the introduction of open source in the life sciences is a reflection of the expression of DNA in a digital or electronic form to be 'acted upon and interacted with in ways that would not otherwise be possible’ (Parry 2004; see also Pottage 65; see also Pottage 2006). Subsequently the second section focuses on synthetic biology, which has in a short period become exemplary for open source as an experimental model and as a more sophisticated approach of genetic engineering.

This part of the article describes in some detail the exchange of synthetic DNA, which sharpens the argument by moving from a focus on “how” knowledge and resources are being shared to a setting that can be reconsider in terms of “who” gets to be a contributer in a field that aims to re-materialize information that is freely available as food, energy, medicine and so forth. More specifically, the introduction of open source in synthetic biology brings together the flexibility in regard of patenting with an increasingly informatic understanding of life and nature. Section two ends with a discussion section on the implications of these limitations on the analogy with open source that is being drawn and signals at the possibility of exploring the realization of a different kind of alternative. Rather than aiming to involve more contributers in experimentation, the analogy could be modeled on the response to the commodification of source code by attempting a much more comprehensive removal of restrictions on DNA, whether as source code, as genetic sequence or as biodiversity, seeds and other kinds of living materials (see Deibel 2006,2009).

## Open source and the convergence of the life sciences and informatics

The language of biological life has become increasingly informationalized since the mid-twentieth century in its emphasis on complexity, adaption, emergence and so on. The dissemination of this informatic paradigm continues today with the integration of molecular genetics and cell biology with fields such as mathematics, statistics and informatics (Doyle 1997; Kay 2000 Fox Keller 2002; O’Malley and Dupré 2007). The emphasis on information is therefore at once a way of thinking that developed throughout the twentieth century and a way of acting revolving around informatic practices and the usage of informatic artifacts. The latter is particularly significant when focusing on the sharing of resources in terms of the 'technological and economic management of information – that is, as a political economy’ (Thacker 2005 Thacker 2003 Haraway 1997 Parry 2004).

Patenting has as its aim to reward inventions by granting exclusive rights that enable temporary monopolies that make it possible to recover the required investments (see Rutz 2009). This includes the life sciences where patenting was introduced in the early eighties by extending the technical criteria for the patenting of chemical compounds. The life sciences were incorporated into the regime for chemistry where patenting had developed in response to advances and industries related to organic chemistry in the late nineteenth century, including the appearance of research laboratories in corporations (Dutfield 2003).

More specifically the patent regimes for the life sciences can be traced to the ruling of the US supreme court in the early eighties that established that DNA could be a 'technical' subject'. The implication of this decision was that legally speaking certain types of DNA were designated as a 'composition of matter' and a 'product of ingenuity' rather than a 'manifestation of nature' (Parry 2004: 85). Alongside the question whether patents encourage innovation, controversy has remained over the desirability of a patent system in areas like the genetic modification of food, medicine and elsewhere (see van Dooren 2007,2009). Despite of the various and continuous controversy the initial ruling has been followed by numerous others in the US and around the world that expanded the scope of patenting. Amongst these is the patenting of the isolation of DNA in a purified form; as sequenced stretches of DNA (Carolan 2010). This can be considered useful; the main condition is that the DNA was not purified before and it makes no difference that the living form from where the DNA was taken might have already existed for quite a while.

What is significant for this paper, however, is how purified DNA is oftentimes not considered as a 'composition of matter' by analogy to patenting in chemistry but as information analogous to informatics. One of the principle aims of patenting is to ensure the reproducibility of the invention by making information public. Accordingly the invention has to be described in the application and the information is released to the public when the grant expires. However, it is only under certain conditions and with considerable difficulty can informatic practices be identified with DNA that has stable chemical properties (Calvert and Joly 2011 Caulfield 2011). On the one hand, the legal process continues to separate DNA as a “composition of matter” from DNA as information which is not patentable. On the other hand, the life sciences and informatics are no longer as different as scientific domains and industries as they were in the early eighties when patenting was introduced.

Both these interpretations can be applied to a recent case: the invalidation of the patents of Myriad Genetics for two genes with mutations that cause cancer (BRCA1 and BRCA2) by the US supreme court. The company patented these two genes as diagnostic tools for the testing of breast cancer and therefore Myriad can ask higher prices for its tests and restrict access to medical information acquired through these tests. The case got a lot of publicity because of the suggestion that there would be stricter criteria for patenting in the US; what is significant, however, is not only whether or not the legal criteria of patenting are changing but that diagnostic tests, like Myrad's, are quintessentially about the invention and usage of informatic artifacts and related practices. What the case shows is that the patentability of DNA that has been isolated and purified presumes an analogy with chemistry while the tests revolve the identification of gene sequences and expressions of a patient by comparison with information in a database (Caulfield 2011 Rodgers 2010 see Carolan 2010).

More specifically the difference between DNA as chemistry and as information was recognized by the judges. The original ruling by the judge of the District Court had invalidated the patents with the observation that the DNA involved was known through its 'information content, its conveyance of the genetic code'. Similarly the final ruling by the supreme court explains that Myriad's claims are not expressed in terms of chemical composition and did not create or alter the genetic information encoded in the genes or the genetic structure of the DNA^a^ Accordingly Myriad's diagnostic tests are an illustration of the conceptual difficulty and work involved in turning DNA into a biological object that can be patented (Vermeulen et al. 2012). This observation applies to less well-known cases, particularly the attempts to patent models of networks of genes and proteins, which is much more difficult than when patenting a single expression of a gene (Allarakhia and Wensley 2005: 1486). These network patents are sometimes granted in the US and the EU but not many; it is exceedingly difficult to isolate the patentable attributes of networks of genes that are modeled as interactive and related to a wider biological context in its complexity (see Calvert 2007,2008).

Furthermore the practice of presenting an invention based on DNA that is sufficiently chemical to be patenting can be reconsidered as a relationship between the ownership of knowledge and an increasingly complex and fragmented notion of life at a molecular scale that is known by way of an 'information paradigm’ corresponding to a configuration of 'institutions, procedures, instruments, practices and forms of capitalization’ (Rose 2001, 13–15). Its implication is that the legal focus on DNA as a compositions of matter is situated in the context of the convergence of the life sciences and informatics alongside the introduction of open source models. Both the informatic attributes that are considered when a patent application is evaluated and the need for cheap, efficient and high quality access to interactive databases and software reflect a transformation of the usage of DNA in the life sciences.

The remainder of section one has as its objective to show that the introduction of open source models in the life sciences reflects the usage of informatic artifacts – like software, databases, hardware and so on – and related practices like downloading, copying and searching of information around the world via the Internet. What this demonstrates is that there is nothing “idealistic” about discussing open source in the life sciences: there are many examples when including those that are directly equivalent to informatics (open source software, databases and the computing requirements of data-driven research). Crucially, however, the convergence with informatics implies that open source models are integral to the interpretation of processes of gene expression and regulation and sequences that can be cut, spliced and transcribed in different ways. This does not imply that genetic code and source code are, should or will be the same; examining the re-definition of openness shows that the priority given to the digital in experimental settings corresponds to a reconfiguration of ownership, institutions and practices.

### Open source and bio-informatics

When discussing patenting in the life sciences, whether as a critique or its relative merits as a means to encourage innovation, it is important to realize that there are other means whereby to establish ownership over biological objects. For example there are many different kinds of intellectual properties for source code that were introduced for source code around the same time as patenting for genetically modified organisms. Another option, however, is to release the information by using the open licenses that have become increasingly widespread.

When the scope for patenting DNA no longer increases, other kinds of intellectual properties could become more relevant. For instance there are software patents on a processes that make computers run faster, on the interaction with a program or by some other way whereby efficiency has been increased^b^. Similarly source code and information in databases are considered legally equivalent with written text, meaning that they can be copyrighted. However the limitations on the scope of DNA patenting, as in the Myriad case, do not necessarily lead to other kinds of intellectual property or related forms of ownership over knowledge. Another option has become a possibility in line with the response to the extension of intellectual properties to source code in the early eighties.

Specifically, the Free Software Foundation (FSF) has aimed to counter the restrictions on the usage of source code that became possible by extending intellectual property to software development.

Its founder Richard Stalllman started the FSF in the early eighties and most commentators point to his release of the source code that he had programmed himself, which he made available on the condition that those that used it would do the same. When they used his programming, the end-result should be freely available. Notably Stallman could decide to re-interpret his copyright, which does not need to be registered; it is automatic, for novels, newspaper articles, music and at that moment also for the ones and zeros of source code. This is crucial; Stallman did not renounce the copyright that had been introduced but used it for a different purpose. He began using the copyright he had not asked for with the aim of preventing that programmers of source code and users of software would loose the freedom to control part of their own lives.

By creating the General Public License, the GPL, it became possible for Stallman to release the software that he programmed with the aim of enabling others to use, study, copy, modify and redistribute its source code. The aim is to make software 'free' on the basis of four freedoms:

- the freedom to run the program, for any purpose
- the freedom to study how the program works and adapt it to your need;
- the freedom to redistribute copies so you can help your neighbor;
- the freedom to improve the program, and release your improvement to the public, so that the whole
- community benefits^c^.

The GPL was the first of the many different kinds of open licenses that are by now used throughout informatics and in many other domains. The most widespread examples are the well-known Creative Commons licenses that are being used for text, images, audio, video productions, architectural design as well as for software and databases wherein information is stored on maps of genomes, proteomes and metabolomes and so forth. Such open licensing, however, is used in a more pragmatic sense that is closer to the connotations of the term 'open source'. This is a term that was introduced much later, in the nineties, and it emphasizes the efficiency of sharing and collaboration in informatics. It explicitly intended to replace the idea that free software would be available without any costs, which was considered inappropriate in the corporate world (see Berry 2004,2008). Open source does not focus as much on the freedom of the users of software; it placed more emphasis on the technical issues of software development and emphasizes that it is the programmer of source code that owns it. Code is 'property owned by an individual who has the right to control and develop it’ (Berry 2004 Raymond 1999).

Similarly the success of the open source projects is explained by programmers that choose to contribute the source code they wrote to projects that use an open licensing. Under certain conditions such projects are more efficient than other projects; open source and 'closed source’ are then not about freedom but simply different development models that are more or less efficient under different circumstances. In this respect the thousands of projects illustrate efficiency, a technical orientation and economic sense rather than an alternative that is primarily about freedom. This point of view was already part of the discussion over whether the human genome project was already an 'excellent example' of open source in the life sciences (Opderbeck 2004 Allarakhia 2007). The various laboratories around the world used research standards that had to be flexible to integrate their methodologies and some even developed open source software. However, it was also decided by those running the human genome project that open source would be too controversial (Cukier 2003). The databases with sequence information on the human genome were made publicly accessible for everyone without the condition that enforced the sharing of this information in other formats (Sulston and Ferry 2002: 222).

Therefore information on genes could be patented and this might restrict some types of usage of the information in a database. On the one hand, this is not limited to the human genome but counts for most of the incredible amounts of information in various sub-disciplines of the life sciences. For example there are 100 giga bytes of sequenced DNA compiled in the GOLD database alone and similar databases exist for the 100.000 or so human proteins that make up the proteome as well as for spliciomics, metabolomics and so forth^d^. This is information that is available for further research but does not use open licensing. There is, on the other hand, already open licensing for databases with the aim of guaranteeing the future availability of information. For example there are many different kinds of databases that use the 'science commons' license, like the major protein database called the neurocommons and the hapmap^e^. The main aim of these this type of open licensing is to avoid that other intellectual property claims impede the access to the data by other users and to facilitate this usage (see Gitter 2007).

The availability of the information is integral to the interaction of researchers with a wide range of datasets being used in the different sub-disciplines of the life sciences. This includes many examples of open source projects with users around the world like the the bio-versions of programming languages like are bio- java, bio-perl, bio-spice, bio-harvester and bio-lisp. Each of these has its own voluntary or public science support communities like the Open Bioinformatics Foundation. Elsewhere there are numerous projects in bio-informatics that aim for a wide range of open source software tools for the analysis of data. For example there is Bio-SPICE a software tool set that is specially designed for research into the modeling and simulation of 'spatio-temporal processes in living cells’. Its website describes Bio-SPICE by writing: “Bio-SPICE, an open source framework and software tool-set for Systems Biology”^f^.

Finally, these programs illustrate the significance of the ability to rewrite software programs for asking novel biological questions, which is necessary in many different kinds of experiments. The point is therefore not only that there are some software programs in the life sciences that are open source whereas most are not; open source is integral to forms of experimentation that depends on constant coordination of a 'multiplicity of techniques coordinated on an elevated surface (the screen)' (see Mackenzie 2010: 189). Crucially, the introduction of open source in the life sciences is therefore not a feature of patenting or the proliferation of exclusivity. This is an alternative that takes its shape at the intersection with informatics and as a necessary part of questioning the biological, which is too limited in the case of preprogrammed homology searches of databases or software programs that already make information available in the public domain. This difference is significant: it implies a changing relationship between 'openness' and exclusivity that runs throughout the the life sciences and that requires increasingly wide variety of researchers with highly specialized knowledge and different levels of commitment to work together.

### Common-based strategies

Freely available source code and the ability to rewrite the programming of a particular kind of software or database are significant as examples of the re-definition of openness in the life sciences. The various examples that were mentioned are integral to the translation of DNA into informatic formats and can be followed to much more complex research settings wherein massive amounts of data need to be computed and interpreted. Again there are various kinds of open source models involved, which show how the rise of bio-informatics implies that the sharing of information has become a norm and how open source is one of the more efficient way of processing information about life as essentially complex and interactive.

Consider a project called 'folding@home', which is one of Yochai Benkler's counter examples to the suggestion that drug development is too complex, expensive and time consuming for non-commercial projects based on 'common-based strategies' (see Benkler 2006: 83, 351).What is special about this project is that it models the folding of three-dimensional proteins that might cause diseases on the basis of “voluntary” contributions. Its calculations are made by involving millions of volunteers in a internet-based distributed computation projects (ibid). The contributors do nothing more than to install a small client program that runs on unused capacities of their PCs, which enables packages of data to be sent periodically. However the project is not open source; folding@home does not allow for modification of the programming and redistribution of the data by its collaborators, which raises the question whether there is a difference with 'common-based strategies' that do have these characteristics.

Folding@home became faster than any single supercomputer in 2007, surpassing the petabytes and tetraflops of Blue Gene, a supercomputer build by IBM that is specially developed for experiments in the life sciences (Noble 2006: 65–67). These types of computation are needed to create situations and models of life, which requires the processing of enormous amounts of information. Notably Blue Gene runs on Linux, which is 'the key working example' of open source as 'a collective project that has been shared and worked on freely’ (Berry 2004: 80). Therefore supercomputing and hardware are an integral part of the introduction of open source in the life sciences and signal that its scope includes the various types of computational modeling within experimental set-ups. For example the Blue Brain Project, is one of several wherein Blue Gene is used: its objective is to 'to reverse engineer the human brain and recreate it at the cellular level inside a computer simulation' with the aim to develop brain disease treatments^g^. The experimental side to the convergence with informatics foregrounds the specificity of the interface between the life sciences and informatics. The question that is most important when redefining openness as something primarily informatic is about the scope of collaborations. Benkler's interprets the various kinds of informatics projects in the life sciences by pointing out the differences in 'the number of people who can, in principle, participate in a project’, which he explains as: 'inversely related to the size of the smallest scale contribution necessary to produce a usable module’ (Benkler 2006: 101). Accordingly the modularity of a project might be about distributed computers involving millions or smaller scale efforts with fewer contributers that are more specialized. The former applies to examples like folding@home whereas the latter applies to the collaboration in open source software project, which is widely distributed and involves loosely connected individuals who cooperate with each other for a diverse range of motivations wherein contributors can choose what and when to contribute independently of each other (ibid). The modularity of the different project is determined by the total amount of potential contributors, which decreases in magnitude when having to draw in specific type of contributions, either from more motivated or better skilled contributors on some specific component of a project (see Benkler 2002,2006).

On the one hand the emphasis on the modularity of the projects suggests a commons-based strategy for research networks in the life sciences that are in the process of becoming more collaborative, gradual and global as digital environments (Ewan 2004). Such networks revolve around the ability to contribute, which is the objective of many open source project. A simple example might be the ability to track changes in the source code is complimented by repositories for revisions of other kinds of data (see Dietz et al. 2012). On the other hand the open source model can be distinguished as a common-based strategy that revolves around increasingly informatic ways of thinking about life an nature. Such an open source project aims to achieve the objective of finding contributers by prioritizing the biological over the informatic.

A first example is a project called “Open Worm”. Its aim is similar to the other types of software projects that were discussed; it seeks contributers to program a complicated piece of open source software to simulate the behavior of a microscopic roundworm with a relatively low amount of cells. What is significant, however, is the projects tag-line, which equates its efforts with: “building the first digital life form. Open Source”^h^. Such emphasis on the “building of life” is primarily discursive and might be contrasted to many similar attempts to understand cell behavior that do not emphasize the life-like qualities of their models (Figure 1).

The reason, however, that this is a significant example is an indicator at the overlap between open source programming in the life sciences and synthetic biology. This field has quickly become exemplary for its application of open source principles to genetic engineering. So far the discussion has outline the varies types of examples of open source in the life sciences, showing that the alternative to patenting takes a shape that is directly equivalent to open source in informatics. Accordingly the patenting of DNA is not the primary setting for examining the sharing of resources among life scientists; there are various fields that are engaging directly with informatics, which includes open source programming, databases and hardware as integral parts of experimental set-ups.

The implication is not that there will be no patents or the various controversies will be settled; these are likely to intensify as more legal work will be needed to separate DNA as a composition of matter from DNA as an informatic entity. The same applies to how the digital can be prioritized strategically and selectively in relation to life as a technological creation, whether as a medicinal test, a genetically altered crop, a biofuel or otherwise. This is shown in section two: the aim of focusing on synthetic biology is to examine the analogy with open source in the context of the re-materialization of the freely available information. The widening scope for collaboration can be followed from the convergence with informatics to an emerging open source philosophy that is closely related to a business model, to the governance of risk and a notion of openness that is limited to a design process that benefits from the involvement of the users of synthetic DNA.

## Open source as an approach to genetic engineering

Discussion over the introduction of open source in the life sciences increasingly revolves around the field of synthetic biology. Specifically the activities of the BioBricks Foudation are being showcased as an illustration of sharing and collaboration as integral to the aspiration of designing and engineering 'biological parts, devices and systems' for 'useful purposes’ (see Rathenau Institute 2006).

Many of these parts are being patented and are becoming difficult to use, which is one of the principle reasons for the *BioBrick™* Public Agreement. This is an open licensing scheme in the spirit of the General Public License (GPL) introduced by the BioBricks Foundation: 'to make free the sharing of any genetically encoded function that you might already own or make anew' and to ensure 'that the engineering of biology is conducted in an ethical manner to benefit all people and the planet'^i^.

Accordingly a design community is organized around biological parts that are freely available. There is an on-line registry where biological parts, called BioBricks™, are published as modules that have been standardized to perform a biological function and that 'has been engineered to meet specified design or performance requirements’. These parts can then be attached to others in different combinations as 'functional inputs and outputs’ that are being referred to as biological equivalents of sensors, logic gates and actuators (Canton et al. 2008: 787).

Such an approach to genetic engineering has led commentators to recommend a 'mix of patent and open source incentives [that] is most likely to deliver the cheap and abundant part that synthetic biologists (and by implication, the world) needs’ (Henkel & Maurer 2007: 1). In this regard the activities of the BioBricks Foundation are considered as exemplary for the ability to find alternatives to the tragedy of the anticommons by arranging access and guaranteeing 'openness' alongside the proliferation of patenting elsewhere (see also, Rai & Boyle 2007; Kumar & Rai , Kumar & Rai Kumar & Rai 2007)^j^. Furthermore there is widespread admiration for the 'elegance and playfulness of the programming of the hacker culture of software engineering’ (Brent 2004: 1213–1214). Such an analogy with open source seeks an approach to genetic engineering that is comparable to informatics in its 'quest to understand the quantitative biology of function’ wherein it becomes a matter of coding and programming 'design tools’ (ibid, see also Knight 2005).

Crucially, however, it is mainly in a rhetorical sense that the biological parts of the BioBricks foundation can be compared to well-behaved Lego-like entities that are safe and secure according to protocol. Any BioBrick, despite of the emphasis on standardization and modularity, continues to mutate and show variation in ways that could interfere with its performance. A synthetic system could bind to pre-existing pathways of any of its biological parts in unexpected ways. In effect, there is always some amount of the biological complexity that will be left in place; even when there is no interference with its functionality, there will be interactions of multiple genes, feedback loops and so forth (Arkin & Fletcher 2006).

Complexity and lack of understanding do not imply that synthetic biology could not become highly effective in its approach of genetic engineering. What is significant is that the exaggeration spills over from an experimental approach to the legal domain; the open source model is simultaneously a means to attain an exception to patenting and create an interface with the governance of innovation. On the one hand, this is highly desirable: who would object to an open source model that is presented as something ethical with the aspiration to be beneficial to the people and the planet, as its website mentions? On the other hand, there is a closely related business model in synthetic biology that shows the relationship to the usage of informatic practices and artifacts, which was the main argument of section one. Furthermore examining the analogy with open source informatics in detail shows its limitations: while experiments of synthetic biology might become much more collaborative and modular, there are clear limits when considering its open source model as policy advice or a wider variety of restrictions on the usage of DNA that might need to be removed in order to realize its ethical aspirations.

### The exchange of synthetic DNA as a business model

Open source in synthetic biology subscribes to the underlying value of the patent system, which is the acceleration of the development and introduction of novel technologies (see Hilgartner 2012:192). In addition the exchange of synthetic DNA resembles the formation of informatic markets and informatic commodities.

Consider Blue Gene again: this time as a project that combines an implication of Linux with the business model of IBM. Open source models have become so successful in informatics that they are by now an integral part of the competitive strategy of many of its major companies. Even though Linux represents the greatest achievement that open source has been capable of, it is with ease that it has been 'slotted into corporate software production at companies such as IBM, Compaq, Hewlett Packerd, Apple, and Sun Microsystems' (Mackenzie 2005: 72). What this suggests is that the scope of collaboration in the life sciences is at once an experimental setting, focused on the complexity of the modeling of cells, and a feature of the formation of markets for sophisticated hardware, software and other informatic artifacts that are being used throughout the life sciences. This includes the business model of synthetic biology, which is informatic in the sense that the freely available information in databases can be re-materialized into experimental samples that are improved, optimized or altered in respect of one or more of its properties.

Specifically the business model of synthetic biology revolves around the supply of genes and genomes that are standardized on demand. Scientists could do this kind of synthesis themselves but this would be time-consuming and the prices of DNA synthesis are dropping fast. Examples of companies that specialize in putting together long and complicated pieces of synthetic DNA include Blue Heron Technology, Coda Genomics, GeneArt and DNA 2.0 (Bügel et al. 2007Rathenau Institute 2006). The competition of these companies revolves around the efficiency of accessing information and its effective integration into experiments that represent genes and proteins in countless variants in order to identify the most effective one as a digitalized network of reactions.

Accordingly the most publicized experiment of synthetic biology involved an order by the J. Craig Venter Institute for 101 specially designed fragments of synthesized DNA from some of these same companies. These pieces of synthetic DNA were that were put into a yeast cell to be assembled into a 'living bacterial cell based entirely on the synthetically made genome' by the repair mechanisms of yeast as a step towards the ability to create cells that could behave as instructed by the synthetic genome^k^. Most attention has gone to the question whether this experiment has resulted in the first artificial life form; here the observation is merely that the business model of synthetic biology involves very few patents and revolves around freely available information to be re-materialized as experimental samples.

When attempting to be able to 'plug in' energy, food, or health modules into living materials there is no contradiction between the free availability of genetic techniques considering the involvement of the main protagonists of the BioBricks foundation in Codon Devices. Accordingly the centrality of DNA in informatic formats (discussed in section one) can be followed to the founding of this company in 2004 with venture capital that aimed to become a platform for synthetic biology. Notably this has failed: Codon attempted to combine the delivery of synthesized DNA with the design for applications but in 2009 it went out of business. There is little profit in the synthesis of DNA, which is becoming cheaper and it was not successful in finding customers for the design of applications^l^. The failure is significant because it shows that the production process of synthetic biology is not yet effective and how the success of the BioBricks foundation and related experimental set-ups might fill the gap between the exaggerated informatic rhetoric about genetic engineering and the attempts to commercialize the exchange of synthetic DNA.

Prioritizing an open source philosophy for synthetic biology gives a very different impression than when foregrounding how synthetic biologists are attempting to create synthetic modules to be plugged into living materials and to generally expand the scope for turning synthetic DNA into profitable types of products. On the one hand, the business model of synthetic biology can be seen as a production process that is only complete when finding ways to capture profits from whatever synthetic compound is in demand on world markets – plastics, chemicals, oil etc. Currently numerous types of experiments are being done on synthetic equivalents for breaking down toxins, the improvement antibodies as well as bioplastics, biofuels and medicines (See ETC Group 2007a, b). On the other hand, many of the experiments are not directly profit oriented even though they are similarly linked to the cheap and easily accessible samples delivered by the various companies in synthetic biology.

The combination with open source as an approach to genetic engineering foregrounds how effortless a ground-breaking new field might become an inclusive and ethical alternative to patents on DNA and the monopolization of knowledge. An example that is frequently mentioned in this regard is Jay Keasling’s wok on eradicating a parasite that causes malaria. This line of experiments has attracted a lot of publicity for its attempt to identify a synthetic equivalent to a natural compound that is normally extracted from the sweet wormwood plant that is mostly found in northern China. Keasling’s lab was able to isolate the metabolic pathway responsible for its manufacture in the plant and to insert these pathways into yeast to develop the capacity for the large-scale production of the compound. The task was to re-engineer the most efficient artificial pathway (nine genes) that produces artemisinic acid and to convert this, in a few chemical steps, into an effective antimalaria medicine (see Ro et al. 2006). Also this type of experimentations relies on the business model of synthetic biology and even the most humanitarian experiment reflects the exaggeration of the control over the design of genetic constructs that consists of mostly unknown and unstable biological processes. As an approach, however, the focus on engineering pathways and the open source model come together as an approach that seeks to create a design process that requires a much larger involvement and scope for collaboration. This feature of the open source model will be discussed below. The main argument is that the involvement of expects, policy-makers and students is limited by comparison with open source models in informatics. Following a critical discussion of open source as a response to societal concerns over the risks of synthetic biology, a comparison will be drawn with the reputation of of open source for stability an reliability. Considering how the Linux operating system gets much of its reputation from crashing much less than its proprietary counterparts, is it not the least to expect from open source in synthetic biology that there is complete openness about the exaggeration of its capacities?

### Open source and the users of f synthetic DNA

Bio-safety and bio-security governance are topics that are high on the agenda of policy-makers. An increasingly popular response is to advocate greater openness as a response. This includes the open source model of the BioBricks Foundation, which is expected to mobilize the users of synthetic DNA for the detection of risks alongside students contribute to experiments.

Firstly, open source has been proposed as a more efficient approach to the risks that accompany the ability to synthesize the DNA of polio, smallpox, extinct influenza and dangerous pathogens (see ETC Group 2007b). To address such risks a well-known slogan of the open source movement is mobilized: the necessity 'to create and maintain open networks of researchers at every level, thereby magnifying the number of eyes and ears keeping track of what is going on in the world’ (Carlson 2003: 212). The prevention of applications that are intended to do harm becomes a responsibility of a scientific community that collaborates on their design and whose voluntary contributions are considered as more efficient in the detection of misuse than regulation. The detection of mistakes depends on making available documentation and creating a transparent trial-and-error selection in the design process. In brief, openness is expected to remove the need to trust others not to make mistakes or act with malicious intends when using the technology (see Johnson 2005 Schmidt 2008).

Secondly, the priority given to the self-management of risks reflects a generally favorable attitude towards a the ability of users to innovate. Synthetic biology is than one example among others wherein user-innovation is being underestimated by policy-makers and manufacturers of various kinds, which applies to working with DNA alongside open source software and a wide range of physical products (see Von Hippel 2005). The most visible and popular example in this respect is its student competition, called IGEM, which is held around the world and lets students do experiments in synthetic biology. Examples include 'bacteria computing a bit’, e-coli that does not stink but smells like mint, bacteria that move in patters, that detect light, or that light up and takes pictures (Baker et al. 2006).

On the one hand, the competition aims to demonstrate that synthetic biology is something that even students could do and that the biological parts and components are already sufficiently manageable to allow students to come up with novel applications. Accordingly the enrollment of students in experimentation foregrounds the potential to design synthetic constructs on the basis of voluntary contributions. On the other hand the inclusiveness of the design community begins to look different when recalling the business model of synthetic biology and exaggeration of the knowledge about the new forms of life and related applications that are promised and presented to regulators. The limitations to collaboration in the life sciences are not only a question of lowering the threshold to participate in experimentation. The limitations of open source as an approach to working with synthetic DNA are illustrated in the image below, which shows an alternative scenario for open source in the life sciences based on language that is loosely derived from the layout of the website of Ubuntu and has been adapted with Photoshop.

Ubuntu is one of the most used examples of the numerous 'community developed’ Linux-based operating systems^m^. The picture shows an adaption of one of the pages of its website and illustrates the possibilities for an alternative for what 'free' and 'open' signify in the life sciences. Also its text highlights the importance of users in open source projects in informatics, which is also prominent in synthetic biology; its purpose, however, is to show the limitations of an open source approach whose users range from students who are able to contribute to the design of genetic constructs and synthetic biologists who act as regulators (Figure 2).

Comparing the BioBricks Foundation with openness as implemented in the various distributions of Linux such as Ubuntu shows that the former has very few possibilities types of involvement that would lead to different kinds of priorities about what is a stable release and what is not. Certainly the model in synthetic biology and for open source in the life sciences involves different kinds of experts to collaborate and set new standards by sharing information and making their inventions available. It even involves open licensing. However synthetic biology exaggerates its ability to 'cut' and 'paste' biological modules with rigidly standardized properties while its notion of 'the user’ as innovator is minimal when comparing the design process in synthetic biology with open source in informatics.

Even when involving students in experiments and letting professionals guarantee a more efficient and self-regulated detection of mistakes in the design of synthetic constructs, synthetic biology lacks an open process for setting priorities for its designs. This is explained in the text, which has been adapted to foreground the open source detection of errors (here called 'bug' as is customary in programming jargon). Following the example set by the text of Ubuntu highlights that a 'high priority bug’ does not necessarily involve high risks like a lethal virus or bio-terrorism. For example there are many bugst that are important because they have a small impact on a relatively large amount of its users, like mild anxieties over possible bio-safety issues with synthetic biology that are troublesome to many of the users of synthetic constructs that run biological functions like 'energy’, 'medicine’ and 'food’.

### Towards open source as an alternative

There are some signs that there might be a considerable potential for open source models in the life sciences that are not being realized at this moment, neither in synthetic biology or elsewhere. The potential for a more principled alternative can be brought into focus by exploring the societal implications of a overtly pragmatic analogy with open source. Subsequently it becomes possible to suggest a re-orientation, which is to say that it is to soon too present a fully formed comparison to Linux or that has a clear relationship to the many societal concerns in play.

Firstly, the main argument of this paper is twofold: (1) there is a convergence with open source in informatics that ranges from bio-informatics to synthetic biology and (2) the kind of alternative that is taking shape remains limited. These limitations are practical as explained above in the discussion of the priority that could be given to the users of DNA. Furthermore the potential for an alternative can be foregrounded by briefly comparing the potential for a more principled response that would seek to re-configure institutions and interests in support of those who possess the skills, tools and knowledge that are at stake in a world wherein living materials can be translated into informatic formats *and vice versa*.

How could an equivalent to a more principled stance on openness be understood? Such an analogy could begin by revisiting the FSF and its reaction to the commodification of source code, as discussed in section one. This does not imply that there is a fully-formed countermovement in place: it is too early days for any attempt at a comprehensive or definitive overview of the relationship of oepn source to the many issues on related policy-agendas or the social concerns that might benefit from such an alternative. Are there, however, any reasons to presume that there could not be another kind of analogy? What about seeking to mirror the discussion so far: to aim for an analogy with open source that seeks to follow the example set by the FSF in creating a bottom-up, transparent and decentralized movement that eventually might show the kind of innovation that is possible in a design process that does not exclude the priorities of any of its users?

When open licensing was first introduced, it was not clear whether this was legal and there were few companies and governments that were interested. Only in early nineties there began to be widespread recognition of Linux' capacity to innovate. In the decade that followed the success would result in a movement that had made powerful allies who had a strong interest in undermining Microsoft’s position in the software market (see Mackenzie 2005; Deibel 2009). To some extend this situations can already be observe with open source in synthetic biology, which starts out by being heavily invested in the formation of markets for sophisticated hardware, software and other informatic artifacts that are being used in the life sciences. However, the analysis in this paper has simultaneously shown that there can be open licensing at every point at which there are intellectual properties, which suggests that there is a basis for projects that aim to support a notion of open source wherein the user is anyone who wishes to live and work with DNA on their own terms.

Firstly, the pragmatic alternative that is being proposed can be derived from the words of BioBricks protagonist Drew Endy who observes that nobody wants:

'the equivalent of the operating system for wheat in the year 2050 to be running some, you know, horrendous closed code that crashes all the time and I’ve no idea what’s going on’ (on the radio program Futures of Biotech 8)^n^

Obviously he is correct to the extent that nobody would want a crashing operating system for wheat. There is, however, very little to indicate that the kind of operating system being designed in synthetic biology is comparable to the safety and stability that Ubuntu and Linux are known for. The statement signals at the kind of direction that can be expected from a business model of synthetic biology as a re-materialization of the representation of DNA in a 'purely informational form’ – as data or image – [that] will be 'standing in for particular materials resources henceforth to be absent’ (Parry 2004: 65).

What this kind of comparison with open source in informatics overlooks is again how DNA synthesis is a messy and unpredictable process. As the text from the Ubuntu-website that was shown above has warned, it is necessary to be extremely careful in the design of a new version of Ubuntu because: 'regression in a stable release is a catastrophe'. This kind of stability is missing from Endy's wheat, which seems much more analogous to an operating system that is integrated into the hardware without revealing its source code. Certainly the designs are released under an open license, possible the Biobricks license, but Endy's wheat and similar applications are likely to end up running on organic waste or modified mono-crops that are patented as usual with the same companies selling even more chemicals and insecticides than they do now (see Deibel 2013).

In other words, the kind of open source model that is taking shape in synthetic biology might be compared with using a PC's without necessarily knowing its operating system very well. The profitability of the design of synthetic DNA as a commodity – with or without patents on DNA – requires the mobilization of specialized knowledge and commitment of contributers. This kind of involvement is closely tied to the convergence with informatics, which suggests that the instability caused by commodified mono-crops does not fall within the scope where priorities for 'debugging' can be decided upon in in synthetic biology. To put it differently, the really closed source is left in place, which in this case is about how the usage of crops (like wheat) by farmers and plant breeders has become increasingly restricted after plant biotechnologies were developed. Genetic engineering has been a driver of the corporate concentration of multinationals and leads to integration of the commodity chains for crops, plant biotechnologies and chemicals (Kloppenburg 2004 Wield et al. 2010, 349).

Furtherore Endy's operating system for wheat and the complications of establishing who owns a plant and who decides about its usage signals at the relationship between a certain type of openness with the stagnation of the political agenda for environmental policy-making.

Specifically the Convention on Biodiversity (CBD), concluded in 1992, was the first to make explicit that there should be terms and conditions for legitimately searching and using information that is derived from biodiversity and used in the life sciences. At that time the text called for the fair and equitable sharing of the benefits arising out of the utilization of genetic resources (art 1. CBD). This mean that genetic resources came to be considered as valuable and scarce resource that were at once the basic material of the life sciences and a potential source of revenue whereby to realize the basic premise of the CBD: that financial incentives on the destruction of biodiversity should be replaced by ones that encourage conservation (see Reid et al. 1993 Hayden 2003 Hamilton 2006; Hoare & Tarasofsky , Hoare & Tarasofsky Hoare & Tarasofsky 2007; May & Sell , May & Sell May & Sell 2006; Lee & Wilkinson , Lee & Wilkinson Lee & Wilkinson 2007Safrin 2004: 643–647). There appears little potential for negotiations over compensations when there no longer any patents and when the understanding of a reciprocal relationship between science and society is limited to the objective of removing the legal obstacles to innovation.

This is a scenario that appears when embracing a re-definition of openness as more efficient without considering whether the momentum and structure of the intellectual property system need to be used in order to move in a different direction. Initially there might be very little backing for the need for such an alternative; there will be legal complications, governments might prefer a notion of openness that is voluntary, companies would want to see the relationship to a recognizable business model and public institutions will have to be persuaded. However, there might be different kinds of companies and significant interest from institutions that seek new legal arrangements to fit the changing practices in the life sciences. The important point is that open source principles are already being applied to different ways of working with DNA. Therefore the selective introduction of the pragmatically oriented models shows the scope for a more principled approach. The aspiration should be to articulate the possibility of integrating the various responses into a framework that enables advocacy for a more principled position that pro-actively seeks to set conditions that prevent further restrictions on the usage of DNA and aims for an open source model wherein everyone could participate in the collaborative design process and be able to set its priorities.

Despite many hurdles that are much easier to imagine, there are a number of examples already that begin to show the preliminary contours of an alternative. Among these is the 'Biological Open Source’ (BIOS) initiative from Australia. What it does is to make available a package of patented biotechnologies on condition that any follow-up inventions are returned to the common pool. In other words: anyone who builds upon the contributions of others must contribute any improvements that are made back to the other participants. No one that accesses the technologies can enforce intellectual property rights against other members who signed onto the same terms^o^. However a collaborative approach to genetic techniques is only a part of the whole; it still excludes users without technical expertise that might have to live and work with its applications. This limits the appeal of BIOS; such a project ends up appearing inefficient (in line with the rhetoric of the open source movement) in the absence of a more principled position as a generalized response to the commodification of DNA (analogous to the response to the commodification of source code by the FSF).

What is missing in BIOS, as with the BioBricks GPL and Endy's operating system for wheat, are the kind of 'bugs' that cannot be solved by genetic engineering and that require a much more collaborative view of plant breeding. The alternative to 'horrendously closed code' is than to be able to adapt crops to viruses, particular soil types, specific climatic conditions and so forth. To follow BIOS' licensing in this direction might begin by copylefting datasets and genetic circuitries so that their usage is conditional on the support of farmers’ rights (see Kipp 2005Aoki 2008Shrinivas [Bibr CR83][Bibr CR84]). Furthermore the open system for genetic techniques could be integrated with a GPL for germplasm for the breeding of conventional crops within an organizational setting that is truly collaborative (see Deibel 2009see Kloppenburg 2010a, bsee Kloppenburg and Deibel 2011). Detailing such proposals is beyond the scope of this paper but its feasibility should not be dismissed. Given time and effort a new alliance might be created wherein support for collaborative projects refers to an approach that allows anyone to file 'bugs’ on the biological processes, interactions and reactions in laboratories, fields and bodies.

Obviously there are many question that remain. How to involve the various institutions? How to meaningfully turn models into a policy agendas? How to incorporate societal concerns into specific projects? How to prevent the risk of cooperation of this kind of model by those blind to the dangers of innovation, those that are afraid of technology or otherwise? Such questions require careful analysis and experimentation, which would already be a considerable improvement by comparison to the current debate over the merits of openness, which takes an a-priori position on openness as something beneficial. Opening up a debate has to begin with the very attempt to seek an alternative to the devaluation of many of the forms of materiality that are closely linked to societal concerns, particularly those that involve living and working with DNA in embodied form, like biodiversity, seeds, medicines and so on.

## Conclusions

This article ended with a brief observation on 'what open source could be' in the life sciences. The realization of such an alternative depends on many factors that are beyond this paper, which focused in particular on how open source models are only selectively enabled and constrained in their potential as an alternative in the life sciences.

Throughout the paper it has been affirmed that there is increasing flexibility in regard of the patenting of DNA and that this should be interpreted as a feature of the convergence with informatics. This is the main argument of this paper as it sets the stage for any discussion of the potential of open source as well as the shape of its introduction in the life sciences. On the one hand, DNA patents do not extend easily to networks of genes that are understood within biological context that is modeled in its complexity. It is in experimental settings of this kind that the convergence with informatics is distinguishable from a juridical orientation on patenting and possible alternatives. On the other hand the introduction of open source in the life sciences is in the majority of instances an alternative that is only about the efficiency and capacity of collaboration in programming and in experimental set- ups. This was explained in the first part of the paper that focused on the representation of scientific objects as mediated by informatic artifacts, like supercomputer, numerous kinds of software, databases as well as well as of the most sophisticated models of cell behavior. The second part highlighted that the patenting of DNA is not that relevant for the sale and servicing of informatic artifacts, which includes synthetic biology.

The re-definition of openness in the life sciences can be sharpened by looking at the kind of open source philosophy that is involved. Accordingly the success of open source models in the life sciences can vary but this is not solely a question of efficiency; experimentation that does not depend on patents is prioritized within a context wherein on companies are attempting to profit from servicing informatic artifacts or from royalties and performance based payments on the design of whatever biological commodity is in demand – food stuffs, medicine, hormones, oil and so forth. The exaggerated emphasis on accuracy and effectiveness in synthetic biology is instructive. As explained the analogy with open source to the task-oriented biological components in synthetic biology tends to result in an operating system that runs on the kind of programming that is much too unstable, that risk crashing because of the interface with its hardware and its informatic rhetoric.

The example that was used is the student competition for designing BioBricks, which suggests that synthetic biology could be something that is easy to do. The problem, however, is that open source programming is not easy, like Linux is not necessarily that easy to use. It is not the simplest approach that is important, as Linux illustrates; it is only with releases like Ubuntu that Linux has become available for anyone whereas earlier it was difficult to obtain, install and operate for those without the necessary programming skills. Ultimately the potential of open source in the life sciences is not primarily about the design process for synthetic constructs into something simple or more profitable but its potential to change the way wherein its designs are being run on living materials. Not everyone needs to understand and be involved in the most complicated projects of the life sciences but anyone can be involved in setting standards for reliability, safety and (food) security.

Such an analogy could become possible by adapting the conditions that open licenses impose and applying these to the convergence of the life sciences and informatics. The free availability of information is than a first step towards a form of genetic engineering of biological parts and systems that is truly optional within an alternative wherein collaboration is no longer merely an additional feature of the much older dictum that 'nature is the program; we replicated it; we own it; we are it’ (Haraway 1997: 8). Obviously this kind of alternative is not the direction chosen in most of the examples; there is little indication that the simplified notion of well-behaved Bio-Bricks can be redesigned; that a community of scientists using a GPL would exchange the optimization of the reactions of genes and proteins for a chance 'to optimize, enhance and renormalize what counts as biological’ in society (Thacker 2003: 76). While the conclusion is therefore that the cases that were discussed are aiming for self-regulation and prioritizing the removal of any obstacles to the circulation of knowledge and information, there is still a little bit of time; time to begin making the necessary alliances for another kind of alternative.

The principled approach of the Free Software Foundation (FSF) might be followed from a reaction to the commodification of source code in the early eighties to the aim of removing the restrictions on the usage of DNA in the 2010s and 20s whether as source code, chemicals, seeds, biodiversity and so on. This is the challenge of open source in the life sciences: to focus on users not owners, as one of the saying of the FSF puts it. A response to the commodification of DNA that includes anyone with a relation to living materials, like those who grow it as crops, eat it as food, take it as medicine or – by virtue of their bodies – are it.

## Endnotes

^a^See John Conley, The Genomic Law Report. http://www.genomicslawreport.com/index.php/2013/05/01/some-thoughts-on-myriad-after-the-supreme-court-argument & See IP Watch, see http://www.ip-watch.org/2013/04/23/us-supreme-court-may-invalidate-gene-patents-but-create-little-change/ (checked May 2013). See Supreme court opinions. http://www.supremecourt.gov/opinions/12pdf/12-398_1b7d.pdf

^b^Typically, software patents are traced back to the 1981 case of Diamond v. Diehr, as a result of which mathematical algorithms became patentable. Examples are amazon.com’s one-click and priceline.com’s reverse auction, which are business patents (for a discussion see Parry 2004).

^c^See http://www.gnu.org/philosophy/why-free.html (accessed May 2013) also see Moglen 2003and Mako Hill 2005.

^d^See for example, http://www.genomesonline.org, and http://www.hupo.org/, http://www.ncbi.nlm.nih.gov/Genbank and http://www.nlm.nih.gov/news/press_releases/dna_rna_100_gig.html (accessed May 2013).

^e^On the Neurocommons see: http://neurocommons.org/page/Main_Page. On the Hapmap see http://snp.cshl.org/thehapmap.html.en (accessed May 2013).

^f^see http://biospice.sourceforge.net/, http://www.open-bio.org/wiki/Main_Page, (accessed May 2013) .

^g^The Blue Brain project includes open source software development called NEURON (see The Blue Brain Project, http://www.artificialbrains.com/blue-brain-project). The oldest example of a collaborative and distributed network involved in modeling cells is probably the Japanese e-cell project from 1996 (Ewan 2004). Another example that shows the considerable institutional backing of these kinds of networks is the UCSD 'signaling gateway' , which operates by having experts monitor and update two or three molecules each and peer-review each others work on the thousands of protein molecules inside the database. These are called Molecule Pages, which are backed by the journal Nature and include interactive kinds of pre-publication and peer-review; (Signalling Gateway. http://www.signaling-gateway.org/search/ (accessed May 2013) For an overview see Allarakhia and Wensley (2007).

^h^See http://www.openworm.org/ (accessed May 2013).

^i^See http://syntheticbiology.org/ and http://openwetware.org/wiki/The_BioBricks_Foundation (accessed May 2013).

^j^Rai and Boyle point to the patent on protein binding that enables basic algebra and storage capacity – to be useful for molecular computing (Rai & Boyle 2007). Elsewhere Kumar and Rai give more examples of patents on binding proteins that produce genetic regulation mechanisms such as multi-state oscillators, a genetic toggle switch, and an adjustable threshold switch (Kumar & Rai 2007).

^k^See the J.Craig Venter Intitute, http://www.jcvi.org/cms/research/projects/synthetic-bacterial-genome/press-release/ (accessed December 17 2012).

^l^;On the demise of Codon see Hayden & Ledford 2009at: http://www.nature.com/news/2009/090415/full/458818a.html. For more commentary see Rob Carlson, http://www.synthesis.cc/2009/04/on-the-demise-of-condon-devices.html (accessed May 2013)

^m^The model of the image comes from Ubuntu, https://wiki.ubuntu.com/Bugs/HowToFix (accessed May 2013).

^n^See Futures in Biotech 27 – a radio program by Marc Pelletier at: http://www.twit.tv/fib8 (accessed May 2013).

^o^BIOS offers a technology package that includes an alternative method for transferring genes to plants for Monsanto’s Agrobacterium patent (the Agrobacterium-independent TransBacterTM plant transformation system) and an activity color test that visualizes where genes are and what they do (the GUSPLUS). See BIOS, http://www.bios.net (accessed May 2013).

## References

[CR1] Allarakhia M (2007). A Knowledge Perspective of Strategic Alliances and Management of Biopharmaceutical Innovation: Evolving Research Paradigms.

[CR2] Allarakhia M, Wensley A (2005). Innovation and intellectual property rights in systems biology. Nature Biotechnology.

[CR3] Allarakhia M, Wensley A (2007). Systems biology: A disruptive biopharmaceutical research paradigm. Technological Forecasting and Social Change.

[CR4] Aoki K (2008). Seed Wars: Controversies and Cases on Plant Genetic Resources and Intellectual Property.

[CR5] Arkin AP, Fletcher DA (2006). Fast, chap and somewhat in control. Genome Biology.

[CR6] Baker D (2006). Engineering life: building a FAB for Biology. Scientific American.

[CR7] Benkler Y (2002). Coase’s Penguin or, Linux and the nature of the firm. Yale Law journal.

[CR8] Benkler Y (2006). The Wealth of Networks.

[CR9] Berry DM (2004). The contestation of code: a preliminary investigation into the discourse of the free software and open software movement. Critical Discourse Studies.

[CR10] Berry D (2008). Copy, Rip.

[CR11] Boettiger S, Burk DL (2004). Open source patenting. Journal of International Biotechnology Law.

[CR12] Boyle J (2003). The second enclosure movement. Law and Contemporary Problems.

[CR13] Boyle J (2003). Enclosing the genome: what squabbles over genetic patents could teach us. Advanced Genetics.

[CR14] Boyle J (2008). The Public Domain: Enclosing the Commons of the Mind.

[CR15] Brent R (2004). A partnership between biology and engineering. Nature Biotechnology.

[CR16] Bügel H (2007). DNA synthesis and biological security. Nature Biotechnology.

[CR17] Burk D (2002). Open source genomics. Journal of Science and Technology law.

[CR18] Calvert J (2007). Patenting genomic objects: genes, genomes, function and information. Science as Culture.

[CR19] Calvert J (2008). The commodification of emergence: systems biology, synthetic biology and intellectual property. BioSocieties.

[CR20] Calvert J, Joly P (2011). How did the gene become a chemical compound? the ontology of the gene and the patenting of DNA. Social Science Information.

[CR21] Canton B, Labno A, Endy D (2008). Refinement and standardization of synthetic biological parts and devices. Nature biotechnology.

[CR22] Carlson R (2003). The pace and proliferation of biological technologies Biosecurity and Bioterrorism: Biodefence strategy, practice and science.

[CR23] Carolan MS (2010). Mutability of biotechnology patents: from unwieldy products of nature to independent object/s. Theory Culture & Society.

[CR24] Caulfield T (2011). Reflections on the gene patent war: the Myriad battle. Sputnik and Beyond, Clinical Chemistry.

[CR25] Cooper M (2008). Life as Surplus: Biotechnology and Capitalism in the Neoliberal Era.

[CR26] Cukier KN (2003). Open Source Biotech: can a non-proprietary approach to intellectual property work in the life sciences?.

[CR27] Deibel E (2006). Common genomes: open source in biotechnology and the return of common property. Tailoring Biotechnologies.

[CR28] Deibel E (2009). Common Genomes: on open source in biology and critical theory beyond the patent. PhD Dissertation.

[CR29] Deibel E (2013). Open variety rights: reconsidering the commodification of plants. The journal of Agrarian Change.

[CR30] Dietz C, Horn M, Berthold MR (2012). Integrative open-source software für die bildanalyse in der biologie. BioPhotonik.

[CR31] Doyle R (1997). On Beyond Living: Rhetorical Transformations of the Life Sciences.

[CR32] Dutfield G (2003). Intellectual Property Rights & the Life Science Industries: A Twentieth Century History. Ashgate.

[CR33] ETC Group (2007). Extreme Genetic Engineering: an introduction into synthetic biology.

[CR34] ETC Group (2007). Patenting Pandora's Bug: Goodbye, Dolly…Hello, Synthia!.

[CR35] Ewan S (2004). Source code collaborations open windows of opportunity. Genomics & Proteomics.

[CR36] Fox Keller K (2002). Making sense of life: explaining biological development with models, metaphor and machines. Harvard University Press.

[CR37] Gitter DM (2007). Resolving the open source paradox in biotechnology: a proposal for a revised open source policy for publicly funded genomic databases. Houston Law Review.

[CR38] Hamilton CJ (2006). Biodiversity, biopiracy and benefits: what allegations of biopiracy tell us about intellectual property. Developing World Bioethics.

[CR39] Haraway DJ (1997). Modest_Witness@Second_Millennium. FemaleMan©_Meets_OncomouseTM: Feminism and Technoscience.

[CR40] Hayden C (2003). From markets to market: bioprospecting’s idiom of inclusion. American Ethnologist.

[CR41] Hayden EC, Ledford H (2009). A synthetic-biology reality check: Is the abrupt closure of prominent player Codon Devices an omen for the field?. Nature.

[CR42] Heller M (1998). The tragedy of the anticommons: property in the transition from marx to markets. Harvard Law Review.

[CR43] Heller M, Eisenberg R (1998). Can patents deter innovation? The anticommons in biomedical research. Science.

[CR44] Henkel J, Maurer SM (2007). The Economics of synthetic biology. Molecular Systems Biology.

[CR45] Hilgartner S (2012). Novel constitutions? New regimes of openness in synthetic biology. BioSocieties.

[CR46] Hoare AL, Tarasofsky RG (2007). Asking and Telling: Can "Disclosure of Origin" Requirement in Patent Applications Make a Difference?. The journal of World Intellectual Property.

[CR47] Hope J (2008). Biobazaar: The Open Source Revolution and Biotechnology.

[CR48] Hope J (2009). Open source genetics: conceptual framework.

[CR49] Johnson N (2005). Steal this Genome!.

[CR50] Kay LE (2000). Who Wrote the Book of Life? A History of the Genetic Code.

[CR51] Kipp M (2005). Software and Seeds: Open Source methods. First Monday, 10, 9.

[CR52] Kleinman DL, Vallas S, Frickel S, Moore K (2006). Contradiction in Convergence: Universities and Industry in the Biotechnology Field. The New Political Sociology of Science: Institutions, Networks,and Power.

[CR53] Kloppenburg J (2004). First the Seed: The Political Economy of Plant Biotechnology.

[CR54] Kloppenburg J, Desmarais A, Wittman HK (2010). Seed Sovereignty: the promise of Open Source Biology. Food sovereignty: theory, Praxis and Power.

[CR55] Kloppenburg J (2010). Impeding dispossession, enabling repossession: biological open source and the recovery of seed sovereignty. Journal of Agrarian Change.

[CR56] Kloppenburg & Deibel (2011). Open Source Biology and the Recovery of Seed Sovereignty.

[CR57] Knight TF (2005). Engineering novel life. Molecular Systems Biology.

[CR58] Kumar S, Rai A (2007). 'Synthetic biology:the intellectual property puzzle. Texas Law Review.

[CR59] Lee D, Wilkinson R (2007). The WTO after Hong Kong: progress in, and prospects for, the Doha Development Agenda.

[CR60] Lessing L (2001). The Future of Ideas: the fate of the commons in an interconnected world. Randon House.

[CR61] Mackenzie A (2005). The performativity of code: software and cultures of circulation. Theory, Culture & Society.

[CR62] Mackenzie A (2010). Design in synthetic biology. BioSocieties.

[CR63] Mako Hill B (2005). Towards a standard of freedom.

[CR64] May C, Sell SK (2006). Intellectual Property Rights: a critical history.

[CR65] Moglen E (2003). Anarchy triumphant: free software and the death of copyright. First Monday.

[CR66] Noble D (2006). The music of life: biology beyond the genome.

[CR67] O’Malley M, Dupré J (2007). Size doesn’t matter: towards a more inclusive philosophy of biology. Biology and Philosophy.

[CR68] Opderbeck DW (2004). The Penguin’s genome, or Coase and open source biotechnology. Harvard Journal of Law & Technology.

[CR69] Overwalle G (2009). Patent Pools, Clearinghouses, Open Source Models and Liability Regimes.

[CR70] Parry B (2004). Trading the Genome: Investigating the Commodification of Bio-Information.

[CR71] Pottage A (2006). Too much ownership: bioprospecting in the age of synthetic biology. BioSocieties.

[CR72] Rai A, Boyle J (2007). Synthetic biology: caught between property rights, the public domain, and the commons. PLoS Biology.

[CR73] Rathenau Institute (2006). Constructing Life: Early Social Reflecting on the emerging field of synthetic biology.

[CR74] Raymond ES (1999). The cathedral and the bazaar : musings on Linux and open source by an accidental revolutionary.

[CR75] Reichman JH, Uhlir PF (2003). A contractually reconstructed Research commons for scientific data in a highly protectionist intellectual property environment. Law & Contemporary problems.

[CR76] Reid WV, Laird SA, Meyer CA, Gámez F, Sitenfeld A, Janzen DH, Gollin M, Juma C (1993). Biodiversity Prospecting.

[CR77] Ro D (2006). Production of the antimalarial drug precursor artemisinic acid in engineered yeast. Nature.

[CR78] Rogers M (2010). Shifting sands? the intellectual property basis of biotechnology. Medical Innovation & Business.

[CR79] Rose N (2001). The politics of life itself. Theory, Culture & Society.

[CR80] Rutz (2009). Synthetic biology and patents. A European perspective. European Molecular Biology Organization (EMBO) Reports.

[CR81] Safrin S (2004). Hyperownership in a time of biotechnological promise: the international conflict to control the building blocks of life. The American Journal of International Law.

[CR82] Schmidt M (2008). Diffusion of synthetic biology: a challenge to biosafety. Systems and Synthetic Biology.

[CR83] Shrinivas KR (2006). TRIPs, access to medicines and developing nations: towards and open source solution.

[CR84] Shrinivas KR (2006). Intellectual property rights and bio commons: open source and beyond. International Social Science Journal.

[CR85] Sulston J, Ferry G (2002). The Common Thread: a Story of Science, Ethics and the Human Genome.

[CR86] Sunder Rajan K (2003). Genomic capital: public cultures and market logics of corporate biotechnology. Science as Culture.

[CR87] Sunder Rajan K (2006). Biocapital: the constitution of Postgenomic life.

[CR88] Thacker E (2003). What is Biomedia?. Configurations.

[CR89] Thacker E (2005). The Global Genome: biotechnology, politics and culture.

[CR90] van Dooren T (2007). Terminated seed: death, proprietary kinship and the production of (Bio) wealth. Science as Culture.

[CR91] van Dooren T (2009). Banking seed: use and information in the conservation of agricultural diversity. Science as Culture.

[CR92] Vermeulen N, Tamminen S, Webster A (2012). Bio-objects; Life in the 21st Century.

[CR93] Hippel E von (2005). Democratizing Innovation MIT Press.

[CR94] Wield D, Chataway J, Bolo M (2010). Issues in the political economy of agricultural biotechnology. Journal of Agrarian Change.

